# Capital interplays and the self-rated health of young men: results from a cross-sectional study in Switzerland

**DOI:** 10.1186/s12939-015-0167-x

**Published:** 2015-04-18

**Authors:** Gerry Veenstra, Thomas Abel

**Affiliations:** Department of Sociology, University of British Columbia, 6303 N. W. Marine Drive, Vancouver, BC V6T 1Z1 Canada; Institute of Social and Preventive Medicine, University of Bern, Bern, Switzerland

**Keywords:** Switzerland, Self-rated health, Education, Capitals, Capital interplays

## Abstract

**Introduction:**

We apply capital interplay theory to health inequalities in Switzerland by investigating the interconnected effects of parental cultural, economic and social capitals and personal educational stream on the self-rated health of young Swiss men who live with their parents.

**Methods:**

We apply logistic regression modelling to self-rated health in original cross-sectional survey data collected during mandatory conscription of Swiss male citizens in 2010 and 2011 (*n* = 23,975).

**Results:**

In comparison with sons whose parents completed mandatory schooling only, sons with parents who completed technical college or university were significantly more likely to report very good or excellent self-rated health. Parental economic capital was an important mediating factor in this regard. Number of books in the home (parental cultural capital), family economic circumstances (parental economic capital) and parental ties to influential people (parental social capital) were also independently associated with the self-rated health of the sons. Although sons in the highest educational stream tended to report better health than those in the lowest, we found little evidence for a health-producing intergenerational transmission of capitals via the education stream of the sons. Finally, the positive association between personal education and self-rated health was stronger among sons with relatively poorly educated parents and stronger among sons with parents who were relatively low in social capital.

**Conclusions:**

Our study provides empirical support for the role of capital interplays, social processes in which capitals interpenetrate or co-constitute one another, in the intergenerational production of the health of young men in Switzerland.

## Introduction

We investigate whether and how the cultural, economic and social capitals of Swiss parents and the educational stream of their sons influence the self-rated health of the sons. Inspired by the theories of Pierre Bourdieu [1-4], we use the term ‘capital’ to refer to resources for individual action that are socially valued and objects of struggle. For Bourdieu, the most centrally important forms of capital in social life are cultural capital, economic capital and social capital. Cultural capital refers to material and non-material resources that originate in prevailing social systems of values and symbols. An educational credential is an institutionalized form of cultural capital that represents a certificate of cultural competence which confers on its holder a conventional, constant and legally guaranteed value with respect to culture. Cultural tastes and inclinations, lasting dispositions of mind and body that are socially desirable and relatively rare, represent an embodied form of cultural capital. Objectified cultural capital refers to the possession of valued cultural goods such as books, works of art and machines that require specialized cultural abilities to use. The second primary form of capital, economic capital, refers to money and other assets such as property that can be directly converted into money. The third primary form of capital, social capital, refers to resources embedded in relationships of mutual acquaintance or recognition. Access to the capitals held by others effectively serves to augment or enhance an individual’s own stock of cultural and economic capitals.

Previous research indicates that each of these three distinct forms of capital is implicated in health inequalities. For example, the health effects of institutionalized cultural capital and economic capital have been extensively investigated under the rubric of socioeconomic status and health [5-8]. Embodied cultural capital has also received some attention in recent years [9-13] and research on social capital and health is now voluminous [14-16]. For the most part, these studies have documented distinct health effects of the various forms of capital, typically finding that, all else being equal, more capital tends to correspond with better health.

Rather than treating the various capitals as conceptually and empirically distinct determinants of health, we contend that these capitals are regularly interwoven with one another in processes that produce good health. In this regard our study is influenced by Bourdieu’s ideas pertaining to capital conversions as well as recent discussions on the health effects of what are variously called capital interplays, interactions, relations or transformations [17-20]. Put simply, capital interplays refer to social processes wherein capitals interpenetrate or co-constitute one another. We hypothesize that three kinds of capital interplay are potentially pertinent for the generation of good health, namely, capital acquisition interplays, capital transmission interplays and capital multiplier interplays. We examine each of these intergenerational processes as a generator of positive self-rated health in a sample of young Swiss men collected in 2010–2011 during the process of mandatory conscription of eligible male citizens.

### Capital acquisition interplays

Capital acquisition interplays refer to processes whereby one form of capital facilitates the successful acquisition of another form of capital and then good health. Capital acquisition interplays includes processes wherein parental institutionalized cultural capital facilitates acquisition of other forms of parental capital which subsequently influence the health of the children [21]. After establishing a statistical relationship between the educational credentials of parents and the self-rated health of their sons, we use indicators of various cultural, economic and social capitals held by the parents to explicate the health effects of parental education.

Specifically, inspired by previous research that indicates that the number of books in the home is a valid and reliable indicator of cultural capital [22,23], we use number of books in the home to assess the objectified cultural capital possessed by the families in our study. In this regard we hypothesize that higher educated parents are likely to have a relatively large number of books and that these books add to or reflect the stock of cultural capital in the family. If parental institutionalized cultural capital is a guarantor of command of highbrow culture, then possessing books (objectified cultural capital) is a physical representation of familiarity and facility with social practices typical of highbrow (valued and valuable) culture. We hypothesize that this stock of cultural capital subsequently facilitates the health of their sons via associated social practices, such as parents regularly reading aloud to children when they are young or enrolling children in healthful extracurricular activities (e.g., certain kinds of organized sporting activities). In short, number of books in the family, facilitated by parental education, is an indicator of parental cultural resources broadly writ that function to promote the health of their sons.

We use a measure of perceived financial circumstances to assess parental economic capital. We hypothesize that parental education is strongly related to parental economic capital due to the strong links in Swiss society between educational credentials and occupations. We also hypothesize that economic capital in families is associated with the health of the sons for reasons that relate to the material components of health-related social practices, such as healthy dietary practices in the home (e.g., good food is expensive), and to the accessibility of status symbols (e.g., cars, clothes, practices, etc.) that can foster a sense of security and material stability.

We use perceived parental connections to influential people to assess the social capital of the parents. We hypothesize that higher educated parents will tend to have social ties to highly educated others due to the principle of homophily (birds of a feather flock together) and, therefore, will possess relatively influential ties. We also hypothesize that sons who know that they can rely on their parents’ social capital when needed and who are regularly reassured of their own high social status because of the presence of these potent social ties will be relatively likely to report high levels of health as a consequence.

### Capital transmission interplays

Capital transmission interplays refer to processes whereby one person’s portfolio of capitals is used to contribute to or reshape another person’s portfolio of capitals and, accordingly, their health. This includes processes whereby parental capital indirectly influences children’s health via the educational stream of the children [24]. We investigate whether the health effects of parental capitals are explained by the institutionalized cultural capital of the sons by way of four distinct capital transmission interplays.

First, we hypothesize that the sons of parents with higher educational degrees are relatively likely to be in higher education streams, in part because highly educated parents tend to transmit to and inculcate in their children norms and values that lead their children to (seek to) achieve higher educational credentials themselves. Second, we hypothesize that parents with many books in the home are relatively likely to have sons in higher education streams. Here we posit that number of books in the home is an indicator of a broad range of cultural resources representing linguistic abilities more generally that provides sons with valuable skills for succeeding in the educational system. Third, we hypothesize that parents with more economic capital are more likely to have sons in higher education streams. Wealthier families use higher-level schooling for their children as a strategy for transmitting economic capital to their children via means other than inheritance. In addition, in the Swiss education system a large number of children in the higher streams receive extra-curricular teaching requiring economic investment on the part of the parents. Finally, we hypothesize that parents with more social capital are more likely to have sons in higher education streams. Parental interventions in school can help children succeed in the school system and parents often use their social capital for these kinds of interventions. In addition, parents can use their social capital to select schools that will maximize their children’s chances of succeeding scholastically.

### Capital multiplier interplays

Capital multiplier interplays refer to processes whereby the successful application of one form of capital is facilitated by possession of another form of capital, that is, where one form of capital *multiplies* the effect of another. Multiplier interplays include scenarios wherein the health effect of the educational stream of the sons is facilitated or strengthened by capitals held by the parents. First we examine whether the health effect of the educational stream of the sons is conditional upon the education level of the parents, hypothesizing that personal education will be more strongly associated with self-rated health among sons with higher educated parents than among sons with lower educated parents. This could occur because some of the health promoting characteristics of grammar school environments, such the presence of gymnasiums for certain kinds of physical activity, are more efficacious for the health for sons who are already familiar with the activities that occur in them from early childhood experiences with like-educated parents. In addition, sons may be able to apply their own educational capital more effectively in the labour market and elsewhere when the process of doing so has been previously modelled for them by their parents. Second, we examine whether the health effect of the educational stream of the sons is conditional upon the number of books in the home, hypothesizing that personal education will be more strongly associated with self-rated health among sons with parents who are relatively rich in this form of cultural capital. In this regard the presence of books in the home functions as an indicator of prior familiarity with the intellectual content of higher educational stream settings. Third, we determine whether the health effect of educational stream is conditional upon the economic capital held by the parents, hypothesizing that personal education will be more strongly associated with self-rated health among sons with parents who are relatively wealthy. This could occur if personal education is less efficacious for the production of the good health of people who lack the financial background to implement the healthy lifestyle choices common in the social circles inhabited by people of similar educational attainment. Finally, we hypothesize that the health effect of educational stream is more strongly associated with self-rated health among sons from families with relatively more social capital. Social capital is an indicator of familiarity with values and norms prevalent in higher track education settings that in turn could facilitate more efficacious use of the health promoting aspects of higher educational stream settings.

## Methods

### Survey sample

Our study uses data from the Young Adults Swiss Survey (YASS). The YASS was ethically approved by the supervising board of nine members representing the Swiss National Science Foundation and the Federal Statistical Office. Study procedures were also ethically approved by the scientific advisory board of the YASS study. The target population consisted of all Swiss male citizens of conscription age (around nineteen years of age). Data collection was conducted in 2010 and 2011 at the six national recruiting centres in Switzerland where recruitment for compulsory military service occurs. Participation in the cross-sectional survey was voluntary and anonymous. In total, 31,424 young Swiss men, approximately 40% of the entire population of Swiss men in this cohort, participated in the YASS study. The survey was administered as a paper-and-pencil questionnaire in a classroom setting and was available in German, French and Italian. The survey was administered by non-military staff and collection and processing methods were not linked to the actual conscription procedure. We restricted our investigation to those survey respondents (*n* = 28,795) who lived with one or both parents at the time of the survey. We further restricted our analyses to those respondents (*n* = 23,975) who provided information for all of the variables used in our study. Table 1 describes socio-demographic characteristics of the final sample of survey respondents.

**Table 1:** **Characteristics of the sample**

**Variable**	**Categories**	**n**	**%**
Age	17 years	7	0.0
	18 years	6,424	26.8
	19 years	10,850	45.3
	20 years	4,790	20.0
	21 years	1,323	5.5
	22 years	386	1.6
	23 years	110	0.5
	24 years	68	0.3
	25 years	17	0.1
Immigrant status	born in Switzerland	22,426	93.5
	immigrated to Switzerland	1,549	6.5
Recruitment centre	Lausanne	4,187	17.5
	Sumiswald	3,080	12.9
	MtCeneri	1,437	6.0
	Windisch	7,422	31.0
	Ruti	4,281	17.9
	Mels	3,568	14.9
Highest parental education	mandatory schooling	579	2.4
	vocational	10,588	44.2
	grammar school	1,182	4.9
	technical college	5,171	21.6
	university	6,455	26.9
Personal educational stream	mandatory schooling	1,496	6.2
	vocational	13,824	57.7
	grammar school	8,655	36.1
Number of books in the home	0 to 10	2,026	8.5
	11 to 50	6,864	28.6
	51 to 200	7,426	31.0
	201 to 400	3,934	16.4
	more than 400	3,725	15.5
Parental financial condition	very bad	614	2.6
	bad	4,287	17.9
	good	14,305	59.7
	very good	4,769	19.9
Parental social capital	very unlikely	1,171	4.9
	rather unlikely	3,144	13.1
	rather likely	7,154	29.8
	very likely	12,506	52.2
Self-rated health	poor	63	0.3
	fair	710	3.0
	good	7,483	31.2
	very good	10,949	45.7
	excellent	4,770	19.9

### Survey measures

In Switzerland, approximately one third of adolescents attend grammar schools that lead to university entrance diplomas and then, typically, technical college or university. About six in ten do vocational training in manual or office jobs with one day of vocational school per week. Our respondents were asked about their mother and father’s levels of educational attainment, enabling us to calculate highest parental education attainment. In this regard we distinguish between five levels of parental education: mandatory schooling only, vocational training, grammar school, technical college and university. We use educational stream in three categories to assess the institutionalized cultural capital of the sons, distinguishing between mandatory schooling only, vocational training and grammar school.

To assess the objectified cultural capital of the parents, respondents were asked “How many books are there in your parents’ home?” with response categories ‘none or only a few (0–10),’ ‘enough to fill a book shelf (11–50),’ ‘enough to fill a book case (51–200),’ ‘enough to fill two book cases (201–400)’ and ‘enough to fill three or more book cases (more than 400).’ To assess perceived economic capital, respondents were asked “How financially well-off are your parents?” with response categories ‘very good financial conditions,’ ‘good financial conditions,’ ‘modest financial conditions’ and ‘very modest financial conditions.’ The social capital of parents was assessed by asking respondents “Should you need support from someone in an influential position, someone with connections, could your parents arrange such a contact?” with response categories ‘very likely,’ ‘rather likely,’ ‘rather unlikely’ and ‘very unlikely.’ Consistent with Bourdieu’s understanding of social capital, this measure of social capital, unlike most others in the health determinants literature, focuses on access to the capitals held by others rather than on the presence or number of social ties of any and all kinds.

Global self-rated health, known to encompass both physical and mental well-being and to reliably predict other, more objective, measures of health [25] as well as mortality [26], was assessed as follows: “How would you rate your health status in general?” with response categories ‘excellent,’ ‘very good,’ ‘good,’ ‘fair’ and ‘poor.’

### Statistical methods

Binary logistic regression modelling was applied to self-rated health which we coded poor, fair or good = 0 and very good or excellent = 1. We introduced two-way multiplicative terms to regression models in order to examine statistical interactions between capital variables as predictors of very good or excellent health. Likelihood ratio tests were applied post hoc to assess the statistical significance of the interactions.

## Results

A series of six main effects models on self-rated health are presented in Table 2. Model 1 describes the relationship between parental education and the self-rated health of the sons, controlling for the age and immigrant status of the sons only. In comparison with sons with parents who completed mandatory schooling only, sons with parents who completed grammar school (OR = 1.230, 95% CI = 1.005–1.513), technical college (OR = 1.342, 95% CI = 1.123–1.604) or university (OR = 1.354, 95% CI = 1.136–1.614) were significantly more likely to report better self-rated health scores.

**Table 2:** **Binary logistic regression main effect models on excellent/very good self-rated health**

		**Model 1**		**Model 2**		**Model 3**		**Model 4**		**Model 5**		**Model 6**	
**Variable**	**Categories**	**OR**	**95% CI**	**OR**	**95% CI**	**OR**	**95% CI**	**OR**	**95% CI**	**OR**	**95% CI**	**OR**	**95% CI**
Highest parental education	mandatory schooling (reference)	1.000		1.000		1.000		1.000		1.000		1.000	
	vocational	1.130	0.951..1.344	1.061	0.892..1.264	1.005	0.843..1.197	1.068	0.897..1.271	0.929	0.778..1.110	0.911	0.762..1.988
	grammar school	1.230*	1.005..1.513	1.101	0.892..1.358	1.060	0.860..1.307	1.157	0.939..1.425	0.944	0.762..1.168	0.912	0.736..1.129
	technical college	1.342**	1.123..1.604	1.219*	1.017..1.460	1.107	0.923..1.327	1.217*	1.016..1.456	0.977	0.812..1.175	0.946	0.786..1.139
	university	1.354**	1.136..1.614	1.180	0.984..1.415	1.047	0.875..1.253	1.224*	1.025..1.462	0.904	0.750..1.088	0.861	0.714..1.038
Number of books in the home	0 to 10 (reference)			1.000						1.000		1.000	
	11 to 50			1.195**	1.078..1.324					1.175**	1.058..1.304	1.159**	1.044..1.287
	51 to 200			1.309***	1.180..1.452					1.291***	1.162..1.435	1.255***	1.129..1.395
	201 to 400			1.397***	1.244..1.570					1.343***	1.194..1.512	1.290***	1.145..1.454
	more than 400			1.365***	1.209..1.539					1.287***	1.139..1.455	1.227**	1.083..1.390
Parental financial condition	very bad (reference)					1.000				1.000		1.000	
	bad					1.262**	1.064..1.497			1.214*	1.022..1.441	1.199*	1.009..1.424
	good					1.860***	1.579..2.192			1.701***	1.440..2.009	1.656***	1.402..1.958
	very good					3.073***	2.582..3.659			2.656***	2.223..3.172	2.600***	2.176..3.107
Parental social capital	very unlikely (reference)							1.000		1.000		1.000	
	rather unlikely							1.259**	1.099..1.443	1.153*	1.004..1.323	1.142	0.995..1.312
	rather likely							1.425***	1.257..1.617	1.241**	1.091..1.411	1.239**	1.089..1.410
	very likely							1.881***	1.664..2.126	1.499***	1.322..1.701	1.524***	1.342..1.729
Educational stream	mandatory schooling (reference)											1.000	
	vocational											1.448***	1.448***
	grammar school											1.580***	1.405..1.776
Chi-square (p)		219.82 (<0.001)		259.56 (<0.001)		643.93 (<0.001)		412.65 (<0.001)		751.16 (<0.001)		808.88 (<0.001)	

***p < 0.001, **p < 0.01, *p < 0.05. Each model controls for age and immigrant status. N = 23,975 in each model.

### Capital acquisition interplays

Models 2 through 4 in Table 2 address the degree to which number of books in the home, family economic circumstances and the social capital of parents, respectively, explain the association between parental education and the self-rated health of the sons. These models indicate that, although all three factors are relevant in this regard, economic capital is the more important potentially mediating factor. As a set the three parental capitals explain all of the variability in the self-rated health of the sons attributable to parental education (Model 5).

### Capital transmission interplays

Model 6 in Table 2 indicates that, controlling for the parental capitals, sons in the vocational (OR = 1.448, 95% CI = 1.296–1.617) or grammar school (OR = 1.580, 95% CI = 1.405–1.776) streams were significantly more likely than sons with only mandatory schooling to report better self-rated health. Number of books in the home, perceived financial condition of the family and parental ties with influential others also manifest significant and positive associations with health in this model. Substantially unchanged coefficients for parental capitals from Model 5 to Model 6 indicate that none of the associations between parental capitals and the self-rated health of the sons is explained by the educational stream of the sons. That is, we find little evidence that the transmission of capitals of parents to the institutionalized cultural capital of sons explains variability in the self-rated health of the sons.

### Capital multiplier interplays

Interaction models depicting possible capital multiplier interplays are described in Table 3. For these analyses we dichotomized parental education (university or not), books in the home (more than fifty or fewer than fifty), parental financial condition (very good or not) and parental social capital (very likely or not). Model 1 in Table 3 describes the main effects of the various capital variables on self-rated health while Model 2 describes the interaction between parental education and the educational stream of the sons (the LR test comparing Models 1 and 2 produced *p* = 0.03). Contrary to expectations, personal education was more strongly associated with self-rated health among young men whose parents had less schooling. Among respondents whose parents had not completed university, the odds of sons reporting very good or excellent health were 1.686 (95% CI = 1.469–1.936) times as high for sons in the vocational stream as they were for sons in the mandatory schooling stream and 1.850 (95% CI = 1.590–2.153) times as high for sons in the grammar stream as they were for sons in the mandatory schooling stream. Reproducing Model 2 with parental education reverse coded (model not shown) indicates that the same odds ratios were smaller among respondents whose parents completed university (OR = 1.262, 95% CI = 1.051–1.515 and OR = 1.354, 95% CI = 1.127–1.627, respectively). The interactions between books in the home and educational stream (Model 3) and parental economic capital and educational stream (Model 4) were not statistically significant (*p* > 0.05 in both cases). Model 5 describes the interaction between parental social capital and the educational stream of the sons (*p* = 0.02). Among respondents whose parents had not completed university, the odds of sons reporting very good or excellent health were 1.590 (95% CI = 1.344–1.879) times as high for sons in the vocational stream as they were for sons in the mandatory schooling stream and 1.837 (95% CI = 1.547–2.183) times as high for sons in the grammar stream as they were for sons in the mandatory schooling stream. Reproducing Model 5 with parental social capital reverse coded (model not shown) indicates that these odds ratios were smaller among respondents whose parents completed university (OR = 1.471, 95% CI = 1.271–1.702 and OR = 1.462, 95% CI = 1.252–1.708, respectively).

**Table 3:** **Binary logistic regression interaction models on excellent/very good self-rated health**

		**Model 1**		**Model 2**		**Model 3**		**Model 4**		**Model 5**	
**Variable**	**Categories**	**OR**	**95% CI**	**OR**	**95% CI**	**OR**	**95% CI**	**OR**	**95% CI**	**OR**	**95% CI**
Highest parental education	less than university (reference)	1.000		1.000		1.000		1.000		1.000	
	university	1.027	0.970..1.089	1.357**	1.092..1.686	1.026	0.968..1.087	1.027	0.970..1.089	1.029	0.971..1.091
Number of books in the home	50 or fewer (reference)	1.000		1.000		1.000		1.000		1.000	
	more than 50	1.124***	1.059..1.193	1.124***	1.059..1.193	1.018	0.827..1.252	1.125***	1.060..1.194	1.125***	1.059..1.194
Parental financial condition	less than very good (reference)	1.000		1.000		1.000		1.000		1.000	
	very good	1.684***	1.562..1.817	1.687***	1.564..1.820	1.682***	1.559..1.814	2.023***	1.527..2.681	1.688***	1.565..1.820
Parental social capital	less than very likely (reference)	1.000		1.000		1.000		1.000		1.000	
	very likely	1.324***	1.252..1.400	1.325***	1.253..1.401	1.323***	1.251..1.399	1.323***	1.252..1.400	1.500***	1.214..1.851
Educational stream	mandatory schooling (reference)	1.000		1.000		1.000		1.000		1.000	
	vocational	1.516***	1.385..1.692	1.686***	1.469..1.936	1.458***	1.249..1.703	1.578***	1.399..1.780	1.590***	1.344..1.879
	grammar school	1.636***	1.457..1.836	1.850***	1.590..2.153	1.457***	1.220..1.740	1.671***	1.472..1.896	1.837***	1.547..2.181
Highest parental education*educational stream	less than university and vocational (reference)			1.000							
	university and vocational			0.748*	0.595..0.940						
	less than university and grammar (reference)			1.000							
	university and grammar			0.732*	0.578..0.927						
Number of books*educational stream	50 or fewer and vocational (reference)					1.000					
	more than 50 and vocational					1.083	0.870..1.347				
	50 or fewer and grammar (reference)					1.000					
	more than 50 and grammar					1.202	0.949..1.522				
Parental financial condition*educational stream	less than very good and vocational (reference)							1.000			
	very good and vocational							0.787	0.584..1.059		
	less than very good and grammar (reference)							1.000			
	very good and grammar							0.880	0.647..1.196		
Parental social capital*educational stream	less than very likely and vocational (reference)									1.000	
	very likely and vocational									0.925	0.741..1.155 l
	less than very likely and grammar (reference)									1.000	
	very likely and grammar									0.796	0.632..1.001
Chi-square (p)		665.72 (p < 0.001)		672.70 (p < 0.001)		669.05 (p < 0.001)		669.48 (p < 0.001)		673.65 (p < 0.001)	

***p < 0.001, **p < 0.01, *p < 0.05. Each model controls for age and immigrant status. N = 23,975 in each model.

## Conclusions

Limitations of the study include the fact that each form of capital was assessed by a single indicator. In addition, the measure of cultural capital, assessing number of books in the home, did not account for the kinds of books owned by parents, presumably not all of which can be deemed elements of cultural capital. The measures were based on self-reports, potentially introducing unknown bias in our assessments of individual resources, and the data was cross-sectional, limiting our ability to make definitive causal interpretations. In addition, the study was restricted to a single cohort of young Swiss men, limiting the temporal applicability of our findings. Finally, to the degree that injured or sick men postpone their visits to the conscription centres the prevalence of poor health is underrepresented by our sample. Nonetheless our findings speak to the potentially important role played by the educational system in the intergenerational production and reproduction of social and health inequalities in Switzerland and the relevance of capital interplays for illuminating processes by which this occurs. In particular, our study indicates that capital interplays are central to the successful application of parental institutionalized cultural capital towards achieving the good health of male children in Switzerland.

Consistent with previous research [21,24,27-30], we found that, in comparison with sons whose parents completed mandatory schooling only, sons with parents who completed technical college or university were significantly more likely to report better self-rated health. This result calls for further illumination of the mechanisms by which good health is facilitated by intergenerational social processes. To this end we found that parental economic capital was a potentially important mediating factor in the presumed effect of parental education on the health of their sons, suggesting that material circumstances in childhood are factors in the production of good health among young Swiss men. We also found that number of books in the home manifested an independent association with the health of the young men, suggesting that incorporating book-reading into child-rearing practices and fostering familiarity with cultural symbols of high social status in childhood may be health-relevant forms of embodied cultural capital. Parental social ties to influential people also manifested an independent association with the health and wellbeing of these young men, suggesting that familial access to the capitals of others may be additional resources for the production of good health. Finally, we found that the positive association between educational stream and self-rated health was stronger among young men with lesser educated parents and stronger among young men with parents who were relatively low in social capital. Incompatible with multiplier interplays as we have depicted them here, these results suggest that the health gains stemming from higher-level school environments and/or higher-level educational credentials may partly *compensate* for the social disadvantage that accompanies lower levels of cultural and social capitals in families.

In conclusion, we find empirical support for all of the capital *acquisition* interplays examined in our study, namely, that parental institutionalized cultural capital facilitates the acquisition of parental objectified cultural capital (books in the home), parental economic capital (wealth) and parental social capital (ties to influential people) that then foster the health of young men in Switzerland. We do not find empirical support for any of the capital *transmission* interplays examined in our study, namely, that parental capitals shape the educational stream of the young men and thence their health. Rather, we find that these diverse parental and personal capitals manifest independent associations with the self-rated health of these men. Finally, we find no support for the *multiplier* interplays examined here, namely, that the positive effects of educational stream are stronger for sons with parents possessing more capital of various kinds. Instead, we find that the positive effects of educational stream are stronger for sons whose parents are low in institutionalized cultural capital or low in social capital. We proffer the latter interplays as instances of another kind of capital interplay that can affect health, what we call *compensatory* capital interplays. Future health research in this and other contexts should examine the applicability of this capital interplay as well as the acquisition, transmission and multiplier capital interplays outlined above.
